# What does it cost to redispense unused medications in the pharmacy? A micro-costing study

**DOI:** 10.1186/s12913-019-4065-6

**Published:** 2019-04-24

**Authors:** Charlotte L. Bekker, Helga Gardarsdottir, Antoine C. G. Egberts, Hendrik A. Molenaar, Marcel L. Bouvy, Bart J. F. van den Bemt, Anke M. Hövels

**Affiliations:** 1 Department of Pharmacy, Sint Maartenskliniek, Hengstdal 3, 6574 NA Nijmegen, the Netherlands; 2 Department of Clinical Pharmacy, Division Laboratories and Pharmacy, University Medical Centre Utrecht, Heidelberglaan 100, 3584 CX Utrecht, the Netherlands; 3 Division of Pharmacoepidemiology and Clinical Pharmacology, Utrecht Institute for Pharmaceutical Sciences, Utrecht University, Universiteitsweg 99, 3584 CG Utrecht, the Netherlands; 4 Faculty of Pharmaceutical Sciences, University of Iceland, Sæmundargata 2, 101 Reykjavík, Iceland; 5 Department of Pharmacy, Radboud University Medical Centre, Geert Grooteplein Zuid 10, 6525 GA Nijmegen, the Netherlands; 6 Department of Clinical Pharmacy and Toxicology, Maastricht University Medical Centre, P. Debyelaan 25, 6229 HX, Maastricht, the Netherlands

**Keywords:** Micro-costing, Medication waste, Redispensing, Healthcare economics, Cost-benefit ratio

## Abstract

**Background:**

Redispensing unused medications that have been returned to outpatient pharmacies by patients may reduce waste and healthcare costs. However, little is known regarding the extra costs associated with this process, nor the price level of medications for which this is economically beneficial. The objective of this study was to assess costs associated with redispensing unused medications in the pharmacy and the price level at which redispensing becomes cost-beneficial.

**Methods:**

A micro-costing study was conducted in four Dutch outpatient pharmacies for medications requiring room-temperature storage and requiring refrigeration. First, the pharmacy’s necessary additional process steps and resources for redispensing were identified. Second, time required for each process step was simulated. Third, required resources were quantified by calculating labour, purchasing and overhead costs. Lastly, a model with different scenarios was constructed to calculate the price of a medication package at which redispensing becomes cost-beneficial.

**Results:**

Three main additional process steps for redispensing were identified: (1) pack medications with product quality indicators before dispensing, (2) assess quality of medications returned to the pharmacy (temperature storage, package integrity, expiry date) and (3a) restock medications fulfilling quality criteria or (3b) dispose of medications not fulfilling criteria. Total time required for all steps up to restock one medication package was on average 5.3 (SD ±0.3) and 6.8 (SD ±0.3) minutes for medications stored at room-temperature and under refrigeration, respectively, and associated costs were €5.54 and €7.61. Similar outcomes were found if a medication package would ultimately be disposed of. The price level primarily depended upon the proportion of dispensed packages returned unused to the pharmacy and fulfilling the quality criteria: if 5% is returned, of which 60% fulfils quality criteria, the price level was €101 per package for medications requiring room-temperature storage and €215 per package for those requiring refrigeration. However, if 10% is returned, of which 60% fulfils the quality criteria, the price level decreases to €53 and €109, respectively (arbitrary proportions).

**Conclusions:**

Redispensing unused medications in the pharmacy is at least cost-beneficial if applied to expensive medications.

## Introduction

Pharmaceutical care, including both prescription and over-the-counter medications, represents a substantial proportion of the global healthcare budget [1]. However, up to one-third of patients do, for various reasons, not use all medication dispensed to them [2, 3]. It is difficult to precisely estimate the extent and costs of unused medications because disposal occurs at various moments in time, such as during therapy, months hereafter or even after patient’s death, and through multiple routes, including returning unused medications to the pharmacy, disposing of them as household waste or flushing them down the toilet [4]. Conservative estimates suggest that around $5 billion and £300 million is annually wasted in the US and UK, respectively [2, 5]. These numbers indicate that substantial resources are wasted in the form of unused medications, which highlights the need for the implementation of interventions effectively reducing unnecessary medication waste.

Some packages that are returned to the pharmacy still are completely unopened and intact [6, 7]. These medications could theoretically be redispensed in the pharmacy if they are still of good quality, thereby reducing medication waste and optimising the use of healthcare resources. The discussion on the potential of redispensing unused medications as a waste-reducing intervention is not new [6, 8–10]. However, redispensing is currently not implemented in pharmacies [11], mainly because of legal restrictions, uncertainty about the quality of the returned medications, lack of knowledge regarding patient support for such an approach and uncertainty about the cost-benefits of the redispensing process [12, 13].

To determine whether the implementation of the redispensing of unused medications in the pharmacy is cost-beneficial, a better understanding of the costs associated with this process is required. Such an assessment will facilitate the identification of the types of medications that are eligible for redispensing. This study therefore aimed to assess the costs associated with redispensing unused medications in the pharmacy. Furthermore, an attempt was made to define the price level at which redispensing becomes cost-beneficial.

## Methods

### Study design and setting

A micro-costing study was performed in four hospital-based outpatient pharmacies in the Netherlands between February and June 2016. Micro-costing studies comprise the detailed identification and measurement of all process steps and resources used for an intervention, in this case redispensing, which are subsequently quantified into costs [14]. In this study, a healthcare provider’s perspective was used, for which only the provider’s (pharmacy) costs in the redispensing process were considered. The economic analysis was performed according to Dutch pharmacoeconomic guidelines [15].

An important prerequisite for redispensing unused medications is a guaranteed product quality. To ensure proper storage of medications at patients’ homes, various criteria should be monitored, such as storage temperature, light and humidity exposure and package integrity (unopened, intact). In addition, the medication should have a sufficient long shelf life (here, an expiry date at least 6 months in the future) [10]. It was assumed that an additional outer package (i.e. transparent seal bag) combined with manufacturer’s original primary and secondary packaging would be sufficient to ensure proper storage in terms of light and humidity exposure. This would also facilitate the assessment of the package integrity if the seal is unbroken, ensuring that the package remains unopened and undamaged.

Two types of medications were distinguished based on their storage recommendations; medications requiring storage at room-temperature (15–25 °C) and medications requiring refrigeration at (2–8 °C). Previous research has shown that medications requiring room-temperature storage are generally stored at an appropriate temperature, whereas medications requiring refrigeration are often stored outside the recommended temperature range, including below 0 °C [16, 17]. Therefore, for medications requiring refrigeration detailed temperature information is needed to assess proper storage. It was assumed that a digital temperature measurement logger system would be needed to measure temperature constantly for these medications, but that a simple indicator that indicates out-of-range temperatures (for example, by changing colour) would be sufficient for monitoring storage temperature of medications requiring room-temperature storage.

### Process identification and time measurements

To identify all the additional process steps and resources on top of standard pharmacy practice needed to redispense unused medications, pharmacy staff from the participating four pharmacies were interviewed. The researchers composed a list of the expected process steps and materials required, which was sent to the pharmacists prior to the interview, and the pharmacy staff was asked to adjust the list, adding or excluding steps and materials, and to identify the type of pharmacy staff (e.g. technician or pharmacist) involved in each step.

The identified process steps were simulated in each pharmacy by staff and a researcher recorded the time taken for each step. The simulation was performed three times in each pharmacy. The last simulation in each of the four pharmacies was considered most accurate and therefore used in the analysis (see Table 1 for the process steps and time). Process steps that differed between medications stored at room-temperature or under refrigeration were simulated separately.

**Table 1:** Process steps required to redispense unused medications in the pharmacy, the mean time spent on each step and the associated costs. All process steps could be performed by a pharmacy technician unless stated otherwise

Process steps	Medication requiring room-temperature storage		Medication requiring refrigeration	
	Mean time (minutes; min-max)	Cost (€)	Mean time (minutes; min-max)	Cost (€)
Step 1. Prepare medication before dispensing	1.6 (1.4–1.7)	2.90	2.0 (1.8–2.3)	4.30
▪ Register patient information and medication intended for dispensing in PHIS^a^				
▪ Collect a sealbag and temperature-measuring device				
▪ Activate temperature-measuring device				
▪ Place medication with temperature-measuring device in sealbag				
▪ Inform the patient about the redispensing process				
Step 2. Assess quality of returned medication	2.9 (2.4–3.6)	2.14	3.9 (3.5–4.2)	2.73
▪ Register returned medication in PHIS				
▪ Place medication in a storage location if not assessed directly				
▪ Determine the quality of the medication and register temperature storage, package integrity and expiry date				
▪ Place medication in storage location				
▪ Review and sign off checklist by pharmacist				
Step 3a. Restock medication that fulfil all quality criteria	0.8 (0.6–1.0)	0.50	0.9 (0.8–0.9)	0.58
▪ Collect medication from storage location				
▪ Remove old patient label from medication package				
▪ Document the restocking in PHIS				
▪ Place medication in pharmacy stock^b^				
Step 3b. Dispose of medication that not fulfil quality criteria	0.7 (0.7–0.8)	0.48	0.8 (0.7–1.0)	0.53
▪ Collect medication from storage location				
▪ Document the disposal in PHIS				
▪ Place medication in disposal bin				
Step 4a. Collect and restock temperature loggers returned by post	–	–	0.4 (0.3–0.4)	1.28
Step 4b. Collect and restock temperature loggers returned as normal care			0.4 (0.3–0.4)	0.25
▪ Take logger from envelope (paid by pharmacy)				
▪ Deactivate logger				
▪ Place logger in stock				
Total				
Medication that returns to stock (step 1,2,3a)	5.3 (SD ±0.3)	5.54	6.8 (SD ±0.3)	7.61
Medication that is disposed of (step 1,2,3b)	5.2 (SD ±0.4)	5.52	6.7 (SD ±0.5)	7.56

^a^Pharmacy’s information system, ^b^Stock adjustments and communication with the financial department could not be simulated

### Cost estimation

Direct and indirect costs were calculated for all additional process steps and resources. Direct costs were defined as the pharmacy’s additional costs made during the redispensing process, including labour and materials. Labour costs were calculated for each process step by multiplying the mean time by the costs of the type of pharmacy staff involved, based on the median annual salary reported by the Royal Dutch Pharmacists Association [18]. Salary scales were converted to a per-minute rate based on 1558 working hours per year and a 36-h working week [19]. The salary was increased with 39% to account for social charges [19]. Material costs were calculated using purchase prices. For medications requiring refrigeration, the purchase prices of the digital recording system were included, assuming a life span of 3 years and six uses of the logger. Indirect costs were defined as the pharmacy’s overhead costs made through the employment of staff, the operating activities of the facility and the quality assurance. The overhead costs were valued at 44% of the direct costs [19]. All costs are reported in Euros (2016) and were adjusted using inflation rates where needed [20]. Detailed information on the source of cost information is presented in Table 2.

**Table 2:** Unit cost of labour and materials

Resources	Unit cost (€, 2016)	Source
Pharmacy technician	0.32	Royal Dutch Pharmacists Association
Pharmacist	0.55	Royal Dutch Pharmacists Association
Sealbag	0.42	Transposafe sealbag
Temperature sensor	0.86	Telatemp warmmark time temperature indicator
Temperature logger	10.00	Safe-Rx, Confrerie Clinique
Software and licence for logger	4700.00	Safe-Rx, Confrerie Clinique
Tablet	499.00	Dell-venue 11 pro 7000
Printed paper	0.02	Staples
Printed label	0.01	Zebra Z-select 2000D label
Return envelope	0.72	Dutch post

### Price level

To determine the price level that indicates the price of a single medication package that would be financially eligible for redispensing, a general model was constructed for different scenarios. This model was based on the following assumptions: fixed calculated labour- material- and overhead costs (Table 1), variation in the proportion of medication packages that are returned to the pharmacy (between 1 and 10%) and variation in the proportion of returned medication packages that fulfil all quality criteria (between 20 and 80%). For medications requiring refrigeration, the proportion of loggers that were returned as normal care was set as 77%, the proportion returned by post as 4 and 19% of the dispensed loggers were assumed not to be returned and lost that should be extra purchased (based on personal communication with Vlieland et.al.). To define a base case, the number of medication packages for one therapeutic class dispensed in one pharmacy in 1 year was in this study set as 10,000 (100%). Total costs were calculated and divided by the proportion of returned medication packages that were assumed to fulfil all quality criteria, and as follows the price level for the price of a single medication package was estimated (see Table 3 for example). For estimating the price level the following formula was used:

$$ Price\ level=\frac{Total\ costs\ in\  one\  year}{Proportion\ of\ returned\ medication\ packages\ that\ fulfils\ quality\ criteria\ast 100\%} $$

$$ Total\ costs\ in\  one\  year=\left( cost\ step1\ast 100\% of\ dispensed\ packages\right)+\left( cost\ step2\ast proportion\ of\ returned\ packages\ast 100\%\right)+\left( cost\ step3a\ast proportion\ of\ returned\ packages\ that\ fulfils\ quality\ criteria\ast proportion\ returned\ast 100\%\right)+\left( cost\ step3b\ast proportion\ of\ returned\ packages\ that\ not\ fulfils\ quality\ criteria\ast proportion\ returned\ast 100\%\right)\ast $$

$$ \ast Additional\ for\ medications\ requiring\ refrigeration:+\left( cost\ step4a\ast proportion\ of\ logger s\ returned\  by\  post\ast \left(100\%- proportion\ returned\right)\right)+\left( cost s\ step4b\ast dispensed\ logger s\ returned\  as\  normal\ care\ast \left(100\%- proportion\ returned\right)\right)+\left( cost\ of\ logger s\ lost\ \left[ cost\ logger- cost\ of\ dispensed\ logger\right]\ast proportion\ of\ logger s\ lost\ast \left(100\%- proportion\ returned\right)\right)+ cost\ measuring\ system\ for\  one\  year. $$

**Table 3:** The model to calculate the break-even point, *italic* variables were varied among the scenarios. In this care, 10% of dispensed medication is returned to the pharmacy of which 60% would meet the quality criteria

		Medication requiring room-temperature storage		Medication requiring refrigeration	
	Packages	Cost (€)	Total cost (€)	Cost (€)	Total cost (€)
Step 1.	10,000	2.90	28,978	4.30	43,046
*Step 2.*	*1000 (10%)*	*2.14*	*2138*	*2.73*	*2727*
*Step 3a.*	*600 (60%)*	*0.50*	*298*	*0.58*	*345*
*Step 3b.*	*400 (40%)*	*0.48*	*193*	*0.53*	*210*
Step 4. Loggers	*Of 9000*	–	–		
a. Returned by post	360 (4%)			1.28	462
b. Returned as normal care	6930 (77%)			0.25	1704
c. Lost	1710 (19%)			8.33	14,244
d. Logger system 1 year	1			2496	2496
Total			31,608		65,233
Price level per single package (Total/3a units)			53		109

In addition, the number needed to dispense (i.e. the number of dispensed medication packages that are needed in order to restock one medication package) was calculated for each scenario to indicate the number of medication packages that needed to obtain benefits from redispensing. Therefore, the number of dispensed medication packages was divided by the number of medication packages that returned to stock.

### Data analysis

Data were entered into Microsoft Excel 2010 and descriptively analysed. Averages were expressed as means with standard deviations (SD) or their minimum and maximum values, and proportions were reported as percentages.

## Results

### Process identification and time measurements

**Fig. 1:**
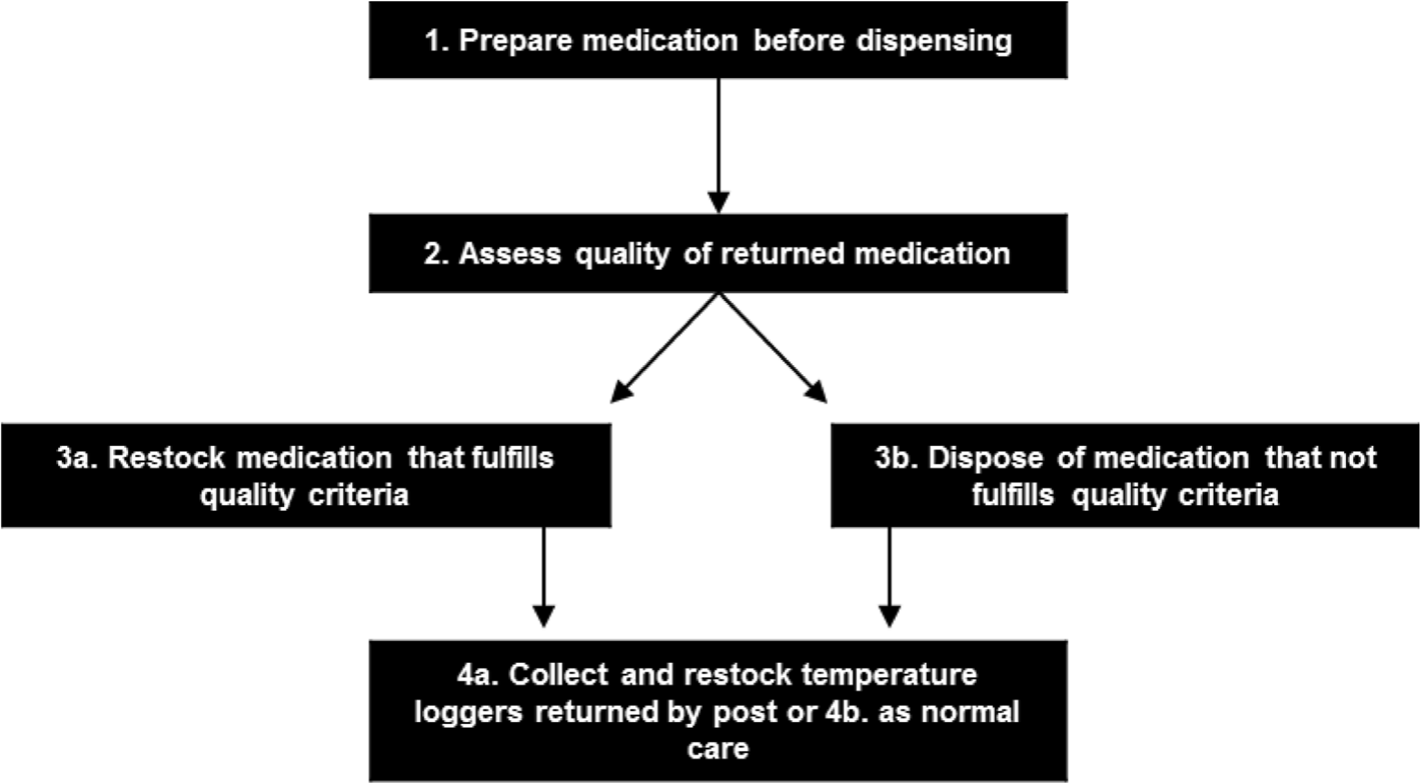
Flowchart of the additional process steps required to redispense unused medications in the pharmacy

To identify the additional process steps and resources required to redispense unused medications in the pharmacy, six interviews were held with eight pharmacists and one pharmacy technician (three interviews were held with two employees simultaneously). During the sixth interview, no new process steps were identified and the composed list was therefore considered comprehensive. Overall, three main process steps were identified in redispensing unused medications in the pharmacy: (1) add materials required for monitoring home storage during the initial dispensing process; (2) assess the quality of the medications returned unused to the pharmacy in terms of temperature storage, package integrity and expiry date; and either (3a) place medications that fulfils all quality criteria into the pharmacy stock or (3b) dispose of medications that not fulfils the quality criteria. As a fourth step for refrigerated medications, patients that use their full medication course would be requested to return the temperature loggers by post (4a) or during their regular pharmacy visit (4b) for reuse. For a general overview of the redispensing process see Fig. 1, and for the process steps see Table 1.

The total time required to perform all process steps up to restocking one medication package was on average 5.3 (SD ±0.3) minutes if requiring room-temperature storage and 6.8 (SD ±0.3) minutes if requiring refrigeration (Table 1). Similar outcomes were found if a medication package would ultimately be disposed of, respectively 5.2 (SD ±0.4) minutes and 6.7 (SD ±0.5) minutes. Time differences between room-temperature stored medications and those requiring refrigeration were a result of time required for temperature logger activation and assessment compared to the temperature sensor. For both medication types, more than half of the time was spent on the quality assessment of returned medications.

### Cost estimation

The costs associated with all process steps and resources, including direct labour- and material costs and indirect overhead costs, required to ultimately return one medication package to stock was €5.54 if requiring room-temperature storage, while these costs were €7.61 for a package requiring refrigeration (Table 1). Similar costs were found if the package would ultimately be disposed of.

### Price level estimation

**Fig. 2:**
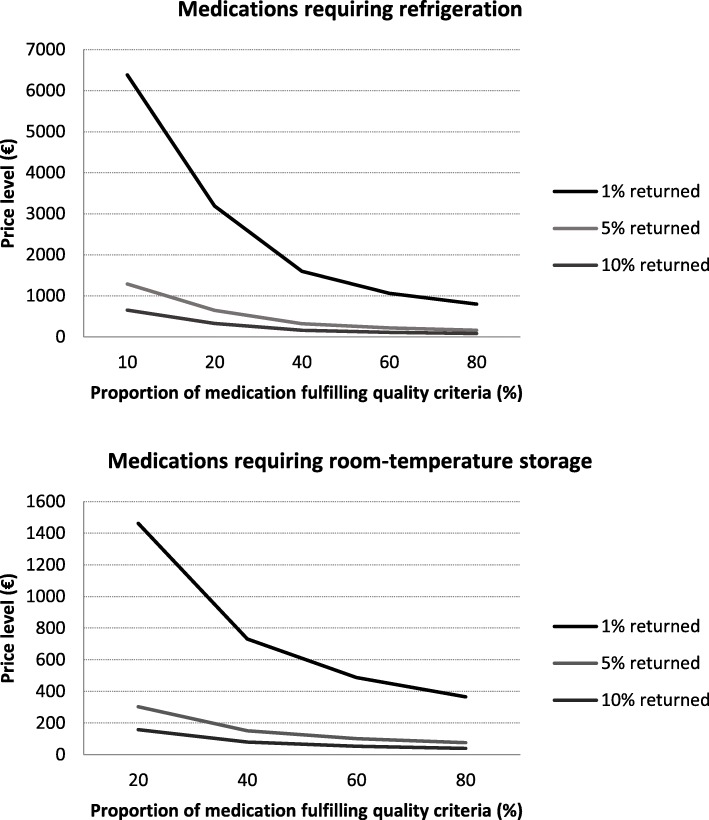
The price level for cost-beneficial redispensing for medications requiring room-temperature storage and refrigeration. The threshold depends on the proportion of dispensed medication packages that are returned to the pharmacy and its proportion that fulfils quality criteria and can be redispensed

The price level of a single returned medication package making redispensing cost-beneficial varied strongly for the different scenarios and decreased when more medications that met the quality criteria would be returned unused to the pharmacy (Fig. 2). For instance, if 5% of the dispensed medication packages would be returned to the pharmacy, of which 60% would fulfil the quality criteria, the price level would be €101.00 per package for medications requiring room-temperature storage and €215.00 for those requiring refrigeration. However, if 10% would return to the pharmacy, of which 60% would fulfil the quality criteria, the price level decreases to €53.00 and €109.00, respectively. Overall, the price level is lower for medications that require room-temperature storage compared to those that require refrigeration.

**Fig. 3:**
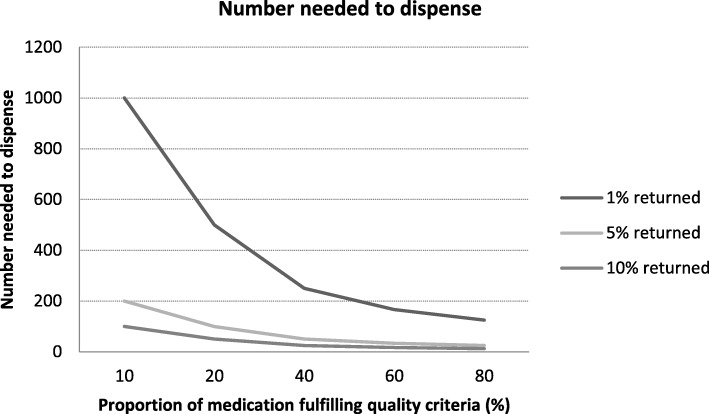
Number of medication packages needed to dispense, which is equally for medications requiring room temperature storage or refrigeration

The number needed to dispense decreased if more medications would return to the pharmacy (Fig. 3). As an example, if 5% would be returned to the pharmacy, of which 60% would fulfil the quality criteria, 33 medication packages needed to be dispensed to allow for restocking of one package.

## Discussion

In this micro-costing study, all additional process steps and resources required to redispense unused medications in the pharmacy were explicitly identified and quantified, and the costs associated with these were assessed. The price-level at which redispensing unused medications becomes cost-beneficial was identified and found to vary strongly depending on the proportion of dispensed packages that is returned unused to the pharmacy that fulfil the quality criteria.

Most studies that address the potential cost savings related to redispensing unused medications include solely the cost of the medications that remain unused [7, 21–24] and do not take into account the associated pharmacy costs. Glanville et al. assessed the pharmacy’s operational costs for redispensing medications donated by patients to patients who lack health insurance and financial means to obtain medication. Their analysis was based on the cost of the donated medications, minus the pharmacy costs needed for the quality assessment, resulting in a total net cost of the redispensed medications [25]. In contrast, this study provides detailed information on the pharmacy costs of the redispensing process when implemented as normal care.

Most costs that enable redispensing would already be made during the initial dispensing of medications to the patient, which requires additional materials to protect the original packaging and to measure home storage temperature conditions. To cover all pharmacy costs associated with redispensing, the price level identified from the analysis indicates that implementation is most likely to be cost-beneficial for expensive medications. The price level was estimated for one therapeutic class of which it was assumed that 10.000 medication packages would be dispensed in a year (base case). However, this may not be feasible to dispense for a small pharmacy. Therefore, a general model was created that can be used for multiple scenarios. Varying the quantity of dispensed medication packages would not impact the determined price levels for medications that require room-temperature storage. However, for medications that require refrigeration, the determined price levels are likely to decrease or increase, due to costs related to the logger system that is needed for monitoring temperature storage. For these reasons also, the price level for cost-beneficial redispensing is higher for medications requiring refrigeration compared to those requiring room-temperature storage.

The price level will decrease if more unused medications that fulfil the quality criteria are returned to the pharmacy. However, many patients do not return their unused medications to the pharmacy and dispose of them through, for instance, the household waste system instead [3, 4]. National awareness campaigns could be implemented to increase the proportion of medication that is returned unused to the pharmacies, which is likely to increase the quantity eligible for redispensing. Increasing patient awareness on proper home storage could also increase the proportion of medications returned to the pharmacy that meet the quality criteria. Campaign costs were not included in this study, and if such awareness programmes were developed, this may affect the estimated process costs if these should be covered by the pharmacy. In addition, implementation costs and effort for large scale workflow adjustments were not considered. Overall, the estimated price levels indicate at which price redispensing becomes cost-beneficial and all costs that the pharmacy makes for this process are covered. In general, large-scale implementation involving more therapeutic classes may decrease the direct and indirect costs of the pharmacy investments per package and could provide further economic benefits.

In this study, the pharmacy’s process costs associated with redispensing unused medications were estimated. If a redispensing system would be implemented in practice as normal care, one should consider how the monetary benefits are shared among all involved stakeholders. It can be argued that patients are less willing to return their unused medications to the pharmacy when only pharmacists financially benefit from redispensing. On the other hand, pharmacists are less likely to redispense medications if the additional costs are not covered. According to stakeholders, the financial benefits can be shared among patients, pharmacists and health insurance companies or used for research [10].

By redispensing unused medications that are currently disposed of, waste can potentially be avoided. In a previous study many stakeholders including pharmacists expressed concerns about the feasibility of implementation of redispensing in current clinical practice [11]. On the other hand, multiple stakeholders highlighted that, in order to realise successful implementation of redispensing, several requirements should be met, such as extensive public engagement, quality assurance of returned medications and an evaluated cost-benefit ratio [10, 13]. Other studies have confirmed that the majority of patients and professionals support redispensing if their concerns about medication safety and quality are addressed [26, 27]. Redispensing is prohibited in some countries under current legislation, mainly due to uncertainty about the quality of unused medications and a fear of counterfeit medications entering the supply chain. The latter is currently being tackled by the European Union Directives 2011/62/EU and EU2016/161, which demand that manufacturers add tamper indicators and unique identification codes to their outer packaging. Furthermore, if these medications are dispensed in a closed seal bag by the pharmacy and only eligible for redispensing when returned unopened this risk is minimised. In terms of quality assurance, medications should be dispensed to patients in the manufacturer’s original outer packaging with tamper-evident seals and thermal devices [13, 28]. Based on these outcomes, one can assume that most requirements to enable the successful implementation of redispensing in practice can be fulfilled.

### Strengths and limitations

The main strength of this study is the use of a micro-costing approach, which is the most comprehensive and precise method to estimate the costs of an intervention [14]. The study also has some limitations. Primarily, this is a simulation study and the process steps that were identified may differ if redispensing is implemented in real practice. However, redispensing is not routinely performed in the pharmacy and therefore these simulations enabled a detailed estimation of the time and resources involved, which was required to calculate the costs. Furthermore, pharmacy staff was not experienced in simulating the process steps, which may have resulted in increased times. Most process steps were similar to the normal pharmacy practice, and three consecutive simulations were performed to increase their experience, of which the last was considered most accurate. It can be assumed that the number of simulations performed by the pharmacy technicians was sufficient to simulate real practice. It was not possible to simulate stock adjustments and communication with the financial department, and no training of the pharmacy staff was included in the analysis. This may have resulted in lower estimations of the time and cost, and ultimately in an underestimation of the price level. However, this would not have altered our general findings that only expensive medication packages are eligible for redispensing. In addition, a healthcare provider’s perspective was used for the cost estimates, and no societal costs were taken into account. In our view, redispensing requires limited effort from society, other than the patients returning their unused medications to the pharmacy. Most patients visit their pharmacy regularly and one can assume that returning unused medication would not result in additional visits. Finally, this study was performed in a Dutch outpatient pharmacy setting and as such, the outcomes may be less generalizable to other countries. We believe that the identified process steps will be similar between countries, however, the unit costs that were included in the analysis may vary. The proportion of dispensed packages that remain unused and are returned to the pharmacy may depend on national prescribing and dispensing policies. Therefore, a general model with various scenarios was build that can be used in different settings as an indicator to determine the price level of medications eligible for redispensing. This model is the best case scenario model and in the real world practice there are some more assumptions and different probabilities which can make these estimated costs higher.

## Conclusions

This study demonstrates that the redispensing of unused medications in the pharmacy is cost-beneficial if applied to expensive medications. This threshold can lower if more medications are returned unused to the pharmacy and have been properly stored at patients’ homes of which the quality can thus be guaranteed.
